# Supplementary material for: A Vertebrate Toxin-Antidote System That Sabotages Mouse Embryogenesis
Source: bioRxiv. 2026 Mar 25:2026.03.04.709565. Preprint. [Version 2] doi: 10.64898/2026.03.04.709565 (PMC13042054; doi:10.64898/2026.03.04.709565)
Supplement: 1 [file NIHPP2026.03.04.709565v2-supplement-1.pdf]

bioRxiv preprint doi: https://doi.org/10.64898/2026.03.04.709565; this version posted March 25, 2026. The copyright holder for this preprint (which was not certified by peer review) is the author/funder. This article is a US Government work. It is not subject to copyright under 17 USC 105 and is also made available for use under a CC0 license.

Supplementary Text

Custom ImageJ/Fiji macro used to quantify 53BP1 foci in Figure 4A:

```
// Perform Z projection, adjust Min and Max, and split channels
run("Z Project...", "projection=[Sum Slices]");
setMinAndMax(1351, 2629);
run("Split Channels");

// Get the list of all open image windows
winList = getList("image.titles");

// Extract the name of the original image
originalImageName = getTitle();  // Get the title of the active image window (before renaming)

// Loop through each window title
for (i = 0; i < winList.length; i++) {
    title = winList[i];

    // Rename windows based on channel
    if (startsWith(title, "C1")) {
        selectWindow(title);  // Make sure to select the window before renaming
        rename("dapi");
    }
    if (startsWith(title, "C2")) {
        selectWindow(title);
        rename("foci");
    }
}

// Now work with the renamed images
selectWindow("dapi");
setAutoThreshold("Otsu dark");
run("Threshold...");
run("Analyze Particles...", "size=100-Infinity circularity=0.60-1.00 display exclude clear
summarize add");

selectWindow("foci");
setThreshold(2250.0000, 1000000000000000000000000000000.0000);
run("Convert to Mask");
run("Find Maxima...", "prominence=100 output=[Single Points]");
roiManager("Show None");
roiManager("Show All");

// Set measurements and analyze
run("Set Measurements...", "area integrated limit redirect=None decimal=3");
run("Clear Results");
roiManager("Measure");
```

bioRxiv preprint doi: https://doi.org/10.64898/2026.03.04.709565; this version posted March 25, 2026. The copyright holder for this preprint (which was not certified by peer review) is the author/funder. This article is a US Government work. It is not subject to copyright under 17 USC 105 and is also made available for use under a CC0 license.

```
// Create a save path using the original image name
savePath = "H:/Embryos/" + originalImageName + "_Results.csv";

// Save results using the original image name
saveAs("Results", savePath);

// Close any window starting with "C2"
for (i = 0; i < winList.length; i++) {
    title = winList[i];
    if (startsWith(title, "C3")) {
        selectWindow(title);
        close();  // Close the selected window
    }
}
selectWindow("Summary");
run("Close");
selectWindow("Threshold");
run("Close");
```

bioRxiv preprint doi: https://doi.org/10.64898/2026.03.04.709565; this version posted March 25, 2026. The copyright holder for this preprint (which was not certified by peer review) is the author/funder. This article is a US Government work. It is not subject to copyright under 17 USC 105 and is also made available for use under a CC0 license.

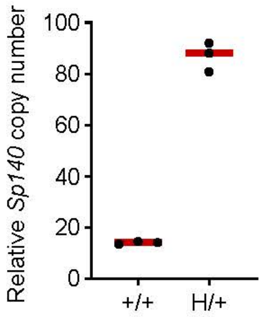

Figure S1 Relative copy number of *Sp140* in *Mus musculus domesticus* estimated by qPCR, related to Figure 2. DNA samples extracted from tail snips were used to examine the copy number increase of *Sp140* gene in H/+ mice compared to +/+. See Method details "DNA isolation and genotyping by qPCR".

bioRxiv preprint doi: https://doi.org/10.64898/2026.03.04.709565; this version posted March 25, 2026. The copyright holder for this preprint (which was not certified by peer review) is the author/funder. This article is a US Government work. It is not subject to copyright under 17 USC 105 and is also made available for use under a CC0 license.

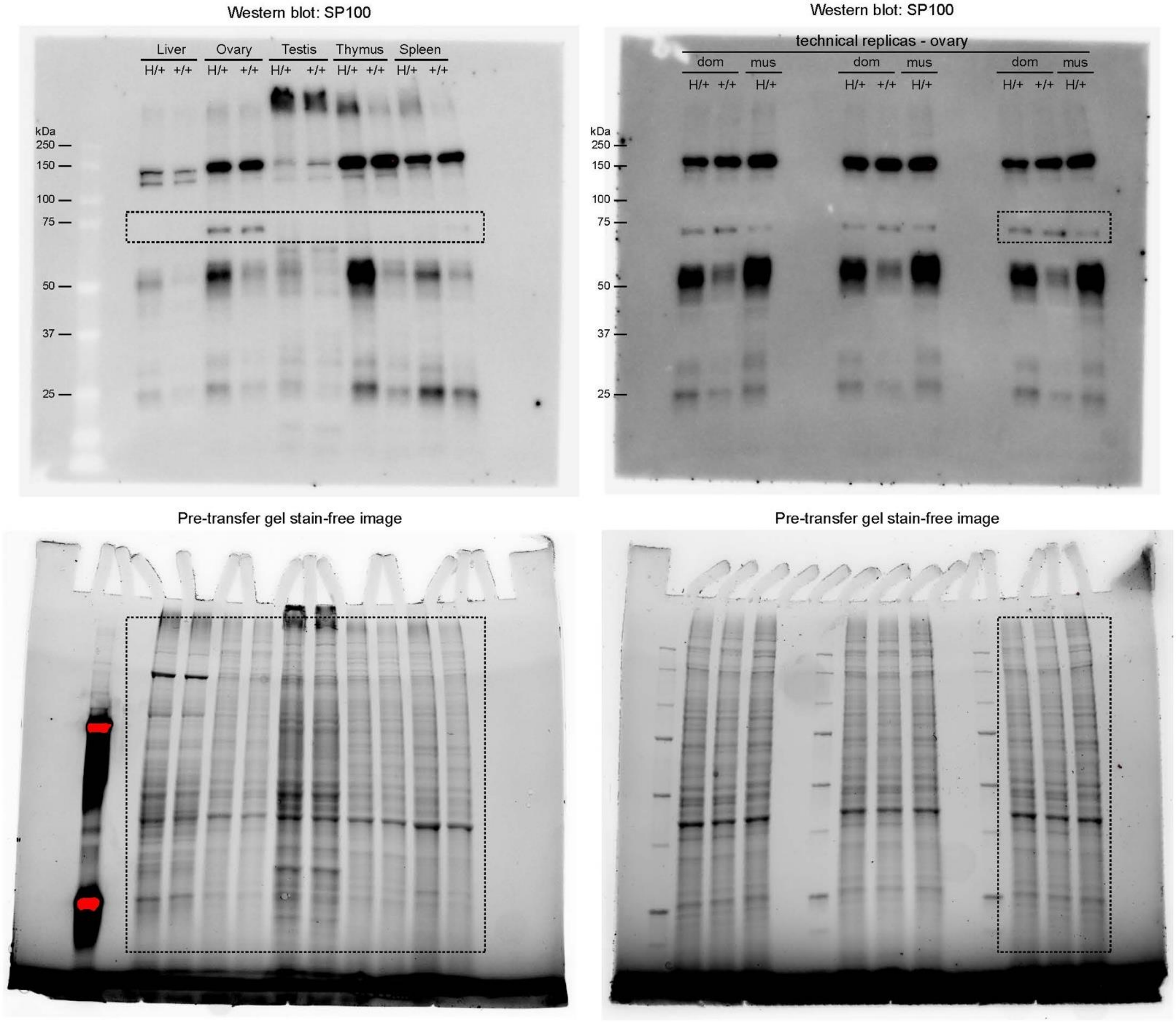

Figure S2 Uncropped images of western blots, related to Figure 2. Entire gels and membranes are imaged. Boxes in the images indicate the cropped area shown in Figure 2G.

bioRxiv preprint doi: https://doi.org/10.64898/2026.03.04.709565; this version posted March 25, 2026. The copyright holder for this preprint (which was not certified by peer review) is the author/funder. This article is a US Government work. It is not subject to copyright under 17 USC 105 and is also made available for use under a CC0 license.

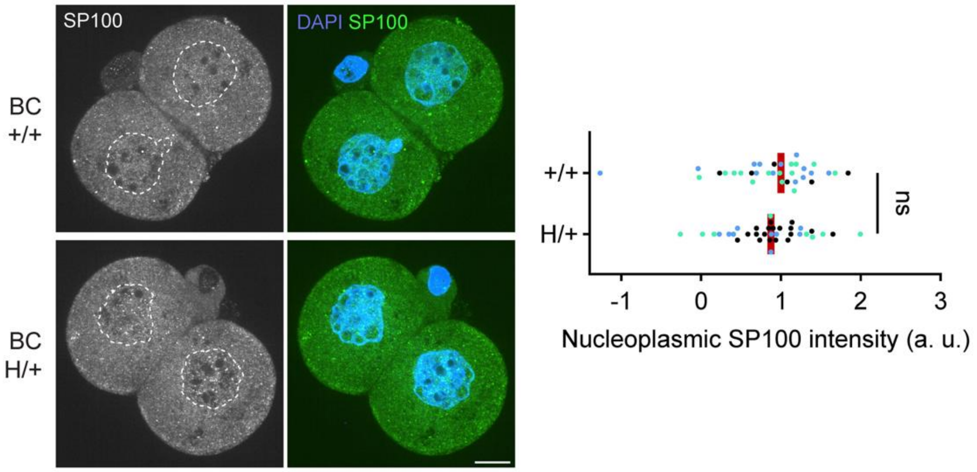

Figure S3 SP100 toxin is equally deposited to +/+ and H/+ embryos, related to Figure 3. Two-cell embryos from BC were fixed, stained for SP100 and genotyped by FISH with the HEX probe. SP100 signal intensities in the nucleus were quantified (n = 33, and 35 for +/+ and H/+, respectively). Mann-Whitney test (two-sided) was used for statistical analysis; ns = 0.5338; red line, median; dots, individual cells. This experiment was repeated independently three times, indicated by dots with distinct colors. Images are optical slices; scale bar, 10 μm.

bioRxiv preprint doi: https://doi.org/10.64898/2026.03.04.709565; this version posted March 25, 2026. The copyright holder for this preprint (which was not certified by peer review) is the author/funder. This article is a US Government work. It is not subject to copyright under 17 USC 105 and is also made available for use under a CC0 license.

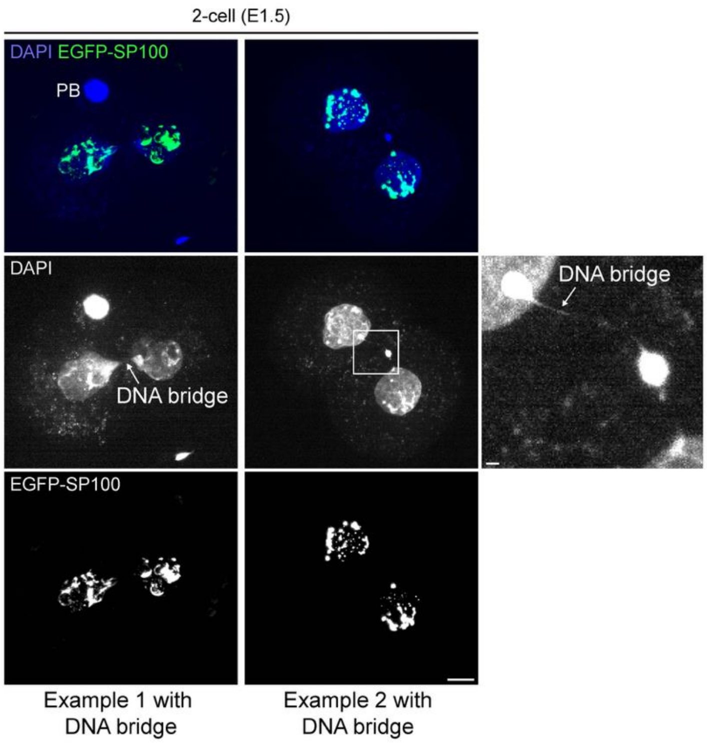

Figure S4 DNA bridges induced upon SP100 overexpression in embryos, related to Figure 4. Embryos overexpressing EGFP-SP100 were fixed at the two-cell stage; Images are maximum intensity Z-projections; E, embryonic day; PB, polar body; scale bar, 10 μm; scale bar of enlarged inset, 1 μm.

bioRxiv preprint doi: https://doi.org/10.64898/2026.03.04.709565; this version posted March 25, 2026. The copyright holder for this preprint (which was not certified by peer review) is the author/funder. This article is a US Government work. It is not subject to copyright under 17 USC 105 and is also made available for use under a CC0 license.

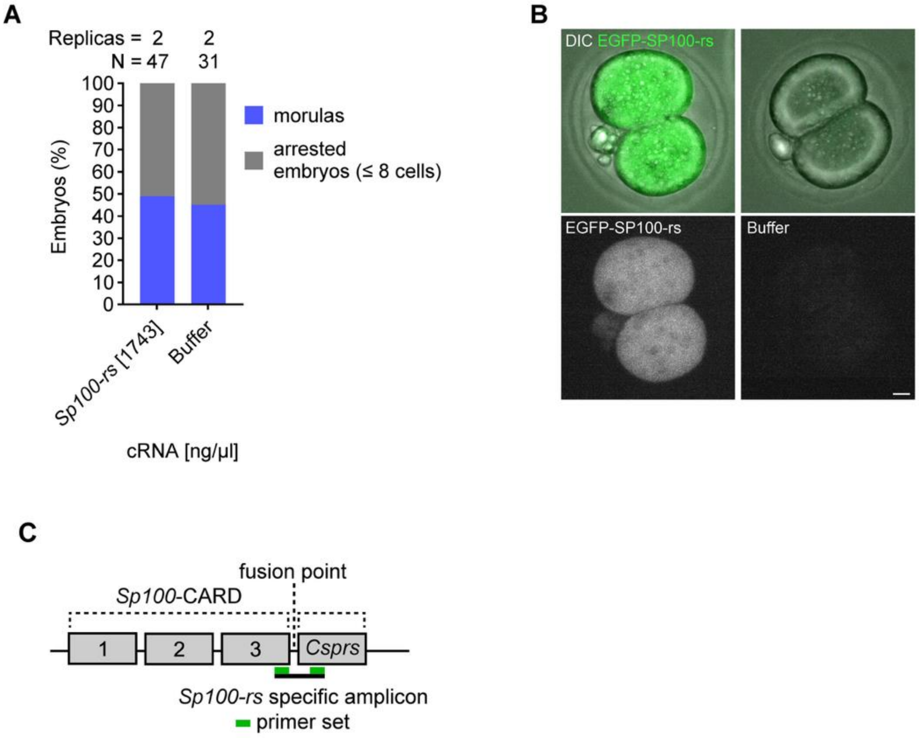

Figure S5 No significant impact on early embryogenesis upon *Sp100-rs* overexpression, related to Figure 4. (A) Control zygotes were microinjected with *Egfp-Sp100-rs* cRNA (1743 ng/µl) and cultured for three days. Embryos were categorized as normal morula (blue) or arrested embryos (grey); sample size is shown above each bar; data from two independent experiments were pooled. **(B)** Control zygotes were microinjected with *Egfp-Sp100-rs* cRNA (1743 ng/µl) or buffer and cultured for one day (the two-cell stage) to examine SP100-rs localization. Ectopically expressed EGFP-SP100-rs uniformly localized in the cell and did not show nuclear enrichment in contrast to SP100 (Figure 4C). Images are maximum intensity Z-projections; scale bar, 10 μm. **(C)** Primer designs to specifically amplify *Sp100-rs*.

bioRxiv preprint doi: https://doi.org/10.64898/2026.03.04.709565; this version posted March 25, 2026. The copyright holder for this preprint (which was not certified by peer review) is the author/funder. This article is a US Government work. It is not subject to copyright under 17 USC 105 and is also made available for use under a CC0 license.

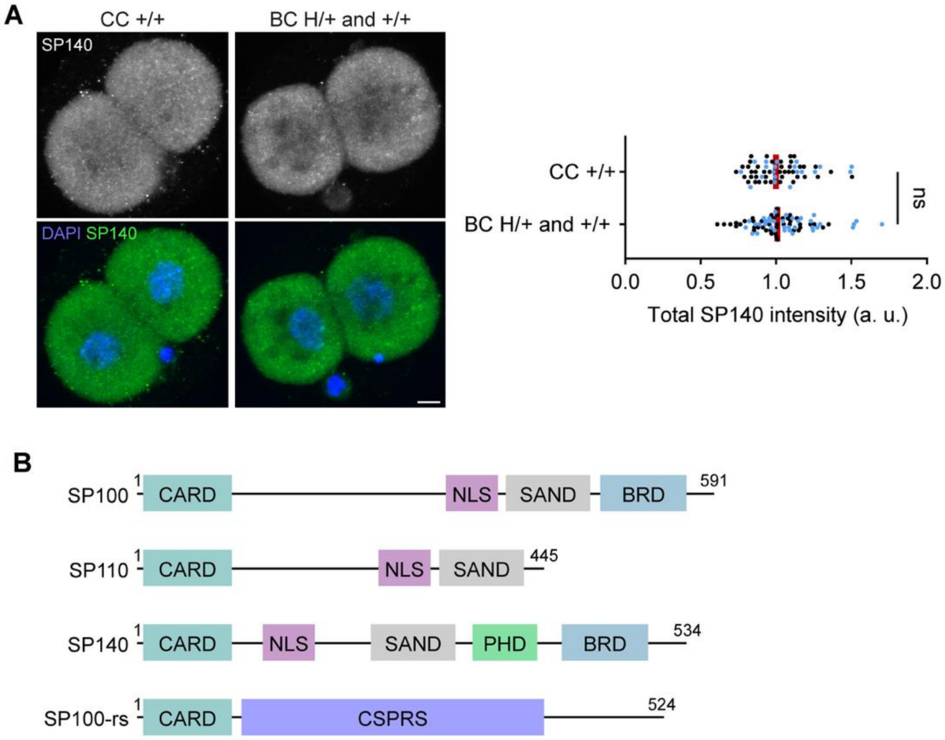

Figure S6 SP140 levels are indistinguishable in two-cell embryos from the control and biasing crosses, related to Figure 4. (A) Embryos from the control cross (CC) and the biasing cross (BC) were fixed at the early two-cell stage and stained for SP140. The signal intensities in the cell were quantified (n = 62 and 78 for +/+ CC and H/+ and +/+ BC, respectively). Mann-Whitney test (two-sided) was used for statistical analysis; ns = 0.7372; red line, median; dots, individual cells from two-cell embryos. Data points were pooled from two independent experiments, indicated by dots with distinct colors. Images are maximum intensity Z-projections; scale bar, 10 μm. **(B)** Domain organizations of the Speckled Protein (SP) family: CARD (Caspase Activation and Recruitment Domain) also known as HSR domain; NLS (Nuclear Localization Signal); SAND (named after its host proteins: SP100, Aire, NucP41/P75, and DEAF); PHD (Plant Homeodomain); BRD (Bromodomain); CSPRS (Component of *Sp100-rs*). SP100-rs does not have NLS, consistent with its uniform distribution in the cell when overexpressed (Figure S5B).

bioRxiv preprint doi: https://doi.org/10.64898/2026.03.04.709565; this version posted March 25, 2026. The copyright holder for this preprint (which was not certified by peer review) is the author/funder. This article is a US Government work. It is not subject to copyright under 17 USC 105 and is also made available for use under a CC0 license.

Table S1.

List of primer sets used in this study.

Assay	Name	Primer (forward)	Primer (reverse)
Genotyping - qPCR	Sp100-rs	TCCACTGAACAGGAGAACACAG	ATGAATTCACGGTCTCGGAGG
	36b4[70]	ACTGGTCTAGGACCCGAGAAG	TCAATGGTGCCTCTGGAGATT
Copy number - qPCR	Sp140	ACAAGGTGGAGATCGCAAGT	GAAGTCTCGGTCTCGAAGGC
Expression - RT-qPCR	Sp100_span	ATCAGCTTGCCGAATCACCA	GTTGAGTTGCACAGCTCTTTCT
	Sp100-rs_span	GAAGCTTTGAATGTGGAGCC	CAGCCAGTCAGATACGTTGC
	Sp110_span	GACATTAAGACATCTGGAGCAGAAAG	AGGCACCCTTCTTTTGAGGT
	Sp140_span	TCGGAGCAGAAGTTTCAGGAAT	TCGTACACGACTCTCTCCACT
	Hprt1_span	CTTCCTCCTCAGACCGCTTT	CATCATCGCTAATCACGACGC
	Rplpo_span	GGACCCGAGAAGACCTCCT	GCACATCACTCAGAATTTCAATGG
	Atp5b_span	GCCACTTCCAAGGTAGCGTT	AGCAACGGTCAAACCAGTCA
	Gapdh_span	GGGTCCCAGCTTAGGTTCAT	CCCAATACGGCCAAATCCGT

bioRxiv preprint doi: https://doi.org/10.64898/2026.03.04.709565; this version posted March 25, 2026. The copyright holder for this preprint (which was not certified by peer review) is the author/funder. This article is a US Government work. It is not subject to copyright under 17 USC 105 and is also made available for use under a CC0 license.

Table S2.

List of the total numbers of events analyzed and the numbers of repeats of each experiment.

Figure 1D. Percentage of embryos genotypes per embryonic day in natural crosses

Experimental group	1.5	4.5	6.5	7.5	8.5	9.5/10.5	11.5
Number of experiments	2	3	> 13	8	4	6	5
Number of embryos	71	44	81	39	27	37	28

Figure 1E. Number of implantation sites in control and biasing crosses per embryonic days

Experimental group	6.5 CC	6.5 BC	7.5 CC	7.5 BC	8.5 CC	8.5 BC	11.5 CC	11.5 BC
Number of females	4	8	3	8	2	5	5	5

* CC +/+ x +/+, BC H/+ x +/+

Figure 1F. Number of resorbing sites in control and biasing crosses per embryonic days

Experimental group	7.5 CC	7.5 BC	8.5 CC	8.5 BC	11.5 CC	11.5 BC
Number of females	3	8	2	4	5	5

* CC +/+ x +/+, BC H/+ x +/+

Figure 1G. Percentage of live embryos after embryo transfer experiment

Experimental group	+/+ 8.5	H/+ 8.5	+/+ 13.5	H/+ 13.5	+/+ 17.5	H/+ 17.5
Number of experiments	1	1	1	1	1	1
Number of embryos	38	25	20	25	24	21

Figure 1H. Percentage of embryo genotypes after embryo transfer experiment and the biasing cross

Experimental group	Transfer	Natural cross
Number of experiments	2	42
Number of samples	51	287

bioRxiv preprint doi: https://doi.org/10.64898/2026.03.04.709565; this version posted March 25, 2026. The copyright holder for this preprint (which was not certified by peer review) is the author/funder. This article is a US Government work. It is not subject to copyright under 17 USC 105 and is also made available for use under a CC0 license.

Figure 2B. Relative mRNA levels of HEX genes in liver - *dom*

Experimental group	+/+ *Sp100*	H/+ *Sp100*	+/+ Sp110	H/+ *Sp110*	+/+ Sp140	H/+ *Sp140*	+/+ *Sp100-rs*	H/+ *Sp100-rs*
Number of experiments	2	2	1	1	1	1	1	1
Number of samples	12	12	5	6	7	6	5	6

Figure 2C. Relative mRNA levels of HEX genes in oocytes - *dom*

Experimental group	+/+ *Sp110*	H/+ *Sp110*	+/+ Sp140	H/+ *Sp140*	+/+ *Sp100-rs*	H/+ *Sp100-rs*
Number of experiments	1	1	1	1	1	1
Number of samples	4	4	7	6	4	4

*20-30 oocytes were pooled for each sample.

Figure 2D. Relative mRNA levels of HEX genes in liver - *mus*

Experimental group	+/+ *Sp100*	H/+ *Sp100*	+/+ Sp110	H/+ *Sp110*	+/+ Sp140	H/+ *Sp140*	+/+ *Sp100-rs*	H/+ *Sp100-rs*
Number of experiments	1	1	1	1	1	1	1	1
Number of samples	5	5	5	5	4	4	5	5

Figure 2E. Relative mRNA levels of HEX genes in oocytes - *mus*

Experimental group	+/+ *Sp110*	H/+ *Sp110*	+/+ Sp140	H/+ *Sp140*	+/+ *Sp100-rs*	H/+ *Sp100-rs*
Number of experiments	1	1	1	1	1	1
Number of samples	5	6	5	6	3	5

*20-30 oocytes were pooled for each sample.

Figure 2F. H3K9me3 signal intensity at the HEX locus

Experimental group	*dom*	*mus*
Number of experiments	2	2
Number of cells	34	51

Figure 3A. SP100 nucleoplasmic signal intensity in oocytes

Experimental group	+/+ control	H/+
Number of experiments	2	2
Number of cells	58	47

bioRxiv preprint doi: https://doi.org/10.64898/2026.03.04.709565; this version posted March 25, 2026. The copyright holder for this preprint (which was not certified by peer review) is the author/funder. This article is a US Government work. It is not subject to copyright under 17 USC 105 and is also made available for use under a CC0 license.

Figure 3B. SP100 total signal intensity in eggs and two-cell embryos

Experimental group	Egg from +/+ +	Egg from H/+ H and +	2-cell from CC +/+	2-cell from BC H/+ and +/+
Number of experiments	2	2	2	2
Number of cells	31	17	100	92

Figure 3B. SP100 nucleoplasmic signal intensity in two-cell embryos

Experimental group	CC +/+	BC H/+ and +/+
Number of experiments	2	2
Number of cells	80	42

Figure 3C. SP110 nucleoplasmic signal intensity in two-cell embryos

Experimental group	+/+ CC	+/+ BC	H/+ BC
Number of experiments	2	4	4
Number of cells	50	47	33

Figure 4A. Percentage of cells with two or more 53BP1 foci per E6.5 embryos from natural crosses

Experimental group	+/+ CC	H/+ BC	+/+ BC
Number of experiments	2	2	2
Number of embryos	24	22	16
Number of cells analyzed per embryo	28-155	22-109	19-149

Figure 4B. Percentage of normal and damaged morulae and arrested embryos after ectopic protein overexpression

Experimental group	Buffer	*EGFP-Sp100* [62.5]	*EGFP-Sp100* [90]	*EGFP-Sp100* [125]	*EGFP-Sp100* [250]
Number of experiments	2	2	2	4	1
Number of embryos	21	22	20	52	7
Experimental group	*EGFP-Sp100* [1767]	*EGFP-Sp110* [125]	*mCherry-Sp110* [125]	*EGFP-Sp140* [125]	*EGFP-Sp140* [250]
Number of experiments	1	2	2	1	2
Number of embryos	13	25	20	7	10

bioRxiv preprint doi: https://doi.org/10.64898/2026.03.04.709565; this version posted March 25, 2026. The copyright holder for this preprint (which was not certified by peer review) is the author/funder. This article is a US Government work. It is not subject to copyright under 17 USC 105 and is also made available for use under a CC0 license.

Figure 4C. EGFP-SP100 nucleoplasmic signal intensity in two-cell embryos

Experimental group	EGFP-Sp100 [90]	EGFP-Sp100 [90] + mCherry-Sp110 [125]
Number of experiments	2	2
Number of embryos	23	24

Figure 4C. Percentage of normal and damaged morulae, and arrested embryos after ectopic protein overexpression

Experimental group	EGFP-Sp100 [90]	EGFP-Sp100 [90] + mCherry-Sp110 [125]	EGFP-Sp100 [125]	EGFP-Sp100 [125] + mCherry-Sp110 [250]	Buffer
Number of experiments	2	2	4	2	2
Number of embryos	20	30	52	32	21

Figure S1. Relative *Sp140* copy number

Experimental group	+/+	H/+
Number of experiments	1	1
Number of samples	3	3

Figure S3. SP100 nucleoplasmic signal intensity in two-cell embryos

Experimental group	+/+	H/+
Number of experiments	3	3
Number of samples	33	35

Figure S5A. Percentage of embryos at each developmental stage after SP100-rs ectopic overexpression

Experimental group	*Sp100-rs* cRNA	Buffer
Number of experiments	2	2
Number of embryos	47	31

Figure S6A. SP140 total signal intensity in two-cell embryos

Experimental group	+/+ CC	H/+ and +/+ BC
Number of experiments	2	2
Number of samples	62	78
